# Beware greedy algorithms

**DOI:** 10.1111/1365-2656.12963

**Published:** 2019-03-15

**Authors:** Benno I. Simmons, Christoph Hoeppke, William J. Sutherland

**Affiliations:** ^1^ Conservation Science Group Department of Zoology University of Cambridge Cambridge UK; ^2^ Faculty of Mathematics University of Cambridge Cambridge UK

## Abstract

To fairly compare the nestedness of ecological networks, a network's observed nestedness can be divided by its maximum nestedness. The authors show that a greedy algorithm does not find networks’ maximum nestedness values. Simulated annealing achieved much better results, laying the foundation for future development of even more sophisticated algorithms.

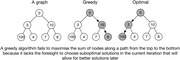

Nestedness—the tendency for specialist species to interact with subsets of the species that generalist species interact with—is a pervasive feature of empirical mutualistic communities (Bascompte, Jordano, Melián, & Olesen, 2003). While theoretical work has discovered important dynamical implications of nestedness, such as enhanced community stability and species coexistence (Bastolla et al., 2009; Rohr, Saavedra, & Bascompte, 2014; Thébault & Fontaine, 2010), there has been less agreement about why networks vary in their levels of nestedness. Answering this question is an important challenge as it has the potential to improve understanding of the mechanisms leading to nested architectures and hence the processes underlying community persistence.

In a recent paper, Song, Rohr, and Saavedra (2017) address this issue in plant–pollinator communities, examining the environmental correlates of nestedness. Specifically, the authors develop a new normalized nestedness metric based on NODF (nestedness based on overlap and decreasing fill) (Almeida‐Neto, Guimarães, Guimaraes, Loyola, & Ulrich, 2008) that, unlike other nestedness measures, can be fairly compared between networks. They use this metric to test the hypothesis that higher levels of nestedness are found in more variable environments, where the enhanced tolerance to environmental perturbations afforded by a nested structure is advantageous. The authors find that their normalized nestedness metric is positively associated with temperature seasonality, supporting this hypothesis, and argue that the lack of relationship in some past studies is due to widespread use of nestedness measures that are not comparable between networks.

This last point is an important one: nestedness is a central concept in the study of mutualistic communities and ensuring it can be compared between networks with different properties is essential. Song et al.'s normalized nestedness metric aims to solve this problem by expressing nestedness as a proportion of the maximum nestedness that can be achieved in a given network with the same number of plants, pollinators and links: NODF_n_ = NODF∕max(NODF). They then additionally control for connectance and network size, as these can also influence nestedness values, to give a final ‘combined nestedness statistic’: NODF_c_ = NODF_n_∕(*C* · log(*S*)), where *C* is connectance and *S* is the geometric mean of the number of plants and pollinators in the network. The authors provide convincing evidence that this statistic is invariant to changes in network size and connectance, unlike alternative normalization methods, such as using *z*‐scores to express nestedness values relative to a set of null expectations.

We welcome the introduction of a new nestedness measure that is comparable across networks, and anticipate it will have wide uptake among network ecologists. However, while we believe the theoretical basis for the combined nestedness statistic is robust, the authors’ proposed method for finding the maximum nestedness that can be achieved in a network with a given number of plants, pollinators and links (with the constraint that all plants and pollinators must have at least one link) has important limitations.

The authors use a greedy algorithm to calculate maximum nestedness. This works by successively adding links to the network, placing each new link in the position that gives the highest NODF value out of all possible positions. An inherent limitation of greedy algorithms is that they lack the foresight to choose suboptimal solutions (in this case, link positions) in the current iteration that will allow for better solutions (higher nestedness values) later in the algorithm. To illustrate this, we consider the following optimization task. Given a graph with a branching structure (see Figure 1a), we want to choose a path from the node in the top level to one of the nodes in the bottom level that maximizes the sum of nodes that lie on the path. Starting from the top‐level node, an intuitive greedy algorithm will proceed by choosing the node with the highest value at each level and will always get stuck in a local optimum (Figure 1b). To achieve the global optimum, it is instead necessary to select the node with the lower value in the middle level, which the greedy algorithm will never do (Figure 1c). One can see that increasing the value of the leftmost node in the bottom level results in an arbitrarily large gap between the optimal and greedy solutions. The limitations of greedy algorithms are well known in other areas of ecology, such as systematic conservation planning, where optimization algorithms are used to solve problems such as “what is the minimum number of sites that need to be designated as protected areas for all species to occur in at least one protected area” or “if a given number of sites can be designated as protected areas, what is the maximum number of species that can occur in at least one protected area?” It was identified early on that greedy algorithms tend to get stuck in local optima when solving these problems (Ardron, Possingham, & Klein, 2010; Ball, Possingham, & Watts, 2009; Kirkpatrick, 1983; Underhill, 1994) and so modern software for these reserve selection tasks, such as Zonation and Marxan, instead uses more sophisticated algorithms to select sites for protection (Moilanen et al., 2005; Possingham, Ball, & Andelman, 2000).

**Figure 1:**
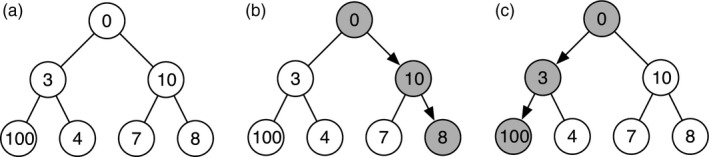
Three graphs with branching structures to illustrate the limitations of greedy algorithms. (a) A graph with a branching structure. We want to select a path from the node in the top level of the graph to one of the nodes in the bottom level, such that we maximize the sum of the nodes that lie on the path. (b) A greedy algorithm would solve this problem by successively selecting the node with the highest value, achieving a sum of 18. (c) The optimal solution, achieving a sum of 103

Without further assumptions, the merit of a greedy algorithm is in speed and simplicity rather than optimality: as we see in Figure 1, the decisions made earlier in the optimization process severely influence the optimization decisions that can be made later on. To demonstrate that the greedy algorithm is unlikely to find the true maximum nestedness of a network, we developed a simulated annealing algorithm. Simulated annealing works by successively applying small modifications to a starting position and accepting or rejecting these based on a temperature parameter. The algorithm derives its name from material science, where annealing describes the process of heating a material, such as glass or steel, and letting it cool down slowly to increase desired properties such as clarity or rigidity. The goal of a simulated annealing algorithm is to abstract from this physical process to create a more versatile optimization algorithm. A classical simulated annealing algorithm requires methods for evaluating the cost and finding the neighbour of a feasible network. In the case of nestedness maximization, we say that neighbours of a given network *web* are those networks *web*′ that can be constructed by moving a single link, and the cost function is given by *web* → 1 − nodf(*web*). A feasible network is one where all plants and pollinators have at least one link.

The simulated annealing algorithm starts with a random feasible network and initializes the current temperature *T* to be the starting temperature *T*_0_. It then proceeds by considering a randomly chosen neighbour of the current network. For both the current network and the neighbour network, the cost function is evaluated:

$$\text{cost}=1-\text{nodf}\left(web\right)\;{\text{cost}}^{\prime }=1-\text{nodf}\left(web^{\prime }\right).$$

If cost′ < cost, we accept the neighbour network as our new solution. If cost′ > cost, we accept the neighbour network with a probability given by Kirkpatrick's acceptance probability function *e* ^cost‐cost′/t^. Consequently, when the temperature *T* is high, the algorithm is more likely to accept suboptimal solutions. In other words, the algorithm is more likely to accept neighbour networks with nestedness values that are lower than the one we have currently found. This allows the algorithm to escape any local optima encountered early in the process and is analogous to random particle movements happening more often in materials with high temperatures. Conversely, when *T* is low, the algorithm is less likely to accept worse solutions, allowing the algorithm to increasingly concentrate on a subset of the solution space where a nestedness value close to the global optimum can hopefully be found.

The algorithm proceeds like this for a given number of iterations *N*, preserving the best solution encountered in case it is optimal. After *N* iterations, the temperature *T* is reduced by multiplying it by a predefined cooling rate 0 < α < 1. Once the temperature falls below a given minimum temperature, *T*_min_, the algorithm terminates, returning the optimal network observed over the entire simulation process. While the above describes the core of our method, the actual algorithm used for our analyses was a modified version of this classical simulated annealing algorithm. The detail of these modifications is given in the Supporting Information. The code for our algorithm is available at https://github.com/CHoeppke/pymaxnodf, alongside a script to reproduce our main result.

We applied our modified simulated annealing algorithm to the same 59 networks analysed by Song et al. Our algorithm found higher levels of maximum nestedness for 58 of these networks (98.3%) (Figure 2). In the one network where our algorithm did not find higher nestedness values (M_PL_042), we achieved a nestedness value equal to the greedy algorithm. This network was the smallest one in the dataset (18 species) and so it is likely that both algorithms found the true optimum nestedness in this case. As can be seen in Figure 2, while for most networks the increase in maximum nestedness was 1% or 2%, for some networks we found substantial increases of up to 17%. This maximum increase of 17% was insensitive to changes in the algorithm parameters (Figure S1). Therefore, normalizing nestedness by dividing the observed nestedness by the maximum nestedness achieved using a greedy algorithm could lead to misleading normalized nestedness values and subsequent ecological interpretations.

**Figure 2:**
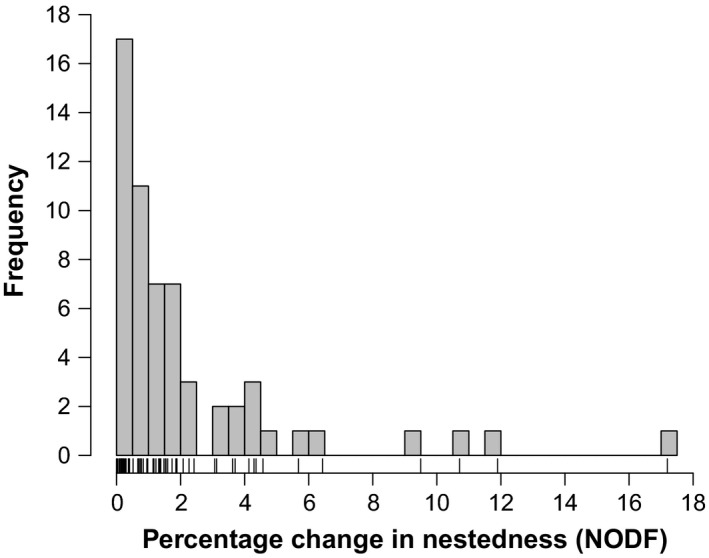
The percentage change in nestedness (NODF) values between Song et al.'s greedy algorithm and our modified simulated annealing algorithm for the 59 networks originally analysed by Song et al. Our algorithm found maximum nestedness values greater than the greedy algorithm in 98.3% of networks and found a value of maximum nestedness equal to the greedy algorithm in the remaining one network

In conclusion, we applaud the combined nestedness statistic introduced by Song et al. The metric fixes a long‐standing problem in network research, and we highly recommend its adoption. We do, however, caution against assuming that a greedy algorithm finds the maximum nestedness of a network. As we have shown here, in 58 of the 59 networks analysed we are able to find a higher (and, in some cases, much higher) maximum nestedness than the greedy algorithm. This is not to suggest that our algorithm found the true global optimum, but rather to prove that the greedy algorithm definitely did not. We therefore need to take on board the lessons learnt from applied mathematics and other areas of ecology, such as systematic conservation planning, and adopt better ways to find the maximum nestedness of a network than a greedy algorithm. We note that adopting more sophisticated algorithms does not necessarily incur any computational cost. Conversely, another benefit of simulated annealing algorithms is that evaluating the NODF metric for neighbour networks can be implemented in a highly efficient way. Consequently, our algorithm improved both the computational and optimization performance of the greedy algorithm. For example, for the network with the most number of links in the dataset (M_PL_015), Song et al.'s greedy algorithm took 33 min to complete, while our algorithm finished this same network in 11 min. We hope this reply will stimulate further research on methods to find the maximum nestedness of networks, with the aim of using Song et al.'s combined nestedness statistic to make nestedness truly comparable between communities.

## AUTHORS’ CONTRIBUTIONS

B.I.S. conceived the study. B.I.S. and C.H. developed the simulated annealing algorithm, conducted analyses and wrote the first manuscript draft. All authors discussed the results, contributed during manuscript writing and approved the final manuscript.

## Data Availability

All data used in this article are available from the open‐access Web of Life repository (http://www.web-of-life.es). Specifically, we used 59 plant‐pollinator networks downloaded from the Web of Life (M_PL_001 to M_PL_059), which can be obtained by going to the Web of Life website, selecting ‘pollination’, then clicking ‘download’.
