# Supplementary material for: Beware greedy algorithms
Source: J Anim Ecol. 2019 Mar 15;88(5):804–7. doi: 10.1111/1365-2656.12963 (PMC6850377; doi:10.1111/1365-2656.12963)
Supplement: Supplementary file 1 [file JANE-88-804-s001.pdf]

Supplementary information

Beware greedy algorithms

Benno I. Simmons[1*†], Christoph Hoeppke[1,2*] and William J. Sutherland[1]

[1] Conservation Science Group, Department of Zoology, University of Cambridge, The David Attenborough Building, Pembroke Street, Cambridge, CB2 3QZ, UK

[2] Faculty of Mathematics, Wilberforce Road, Cambridge CB3 0WA, UK

* These authors contributed equally to this work and should be considered as joint first authors

† Corresponding author: Benno I. Simmons; Conservation Science Group, Department of Zoology, University of Cambridge, The David Attenborough Building, Pembroke Street, Cambridge, CB2 3QZ, UK; benno.simmons@gmail.com

Supplementary Methods

In the main text we detailed the functioning of a classical simulated annealing algorithm. While the classical simulated annealing algorithm was the core of our method, the actual algorithm used for our analyses was a modified version of this algorithm. Here we provide details of these modifications.

Empirical tests showed that the problem of nestedness maximisation is highly susceptible to small changes in networks. This results in cases where high temperatures allow for neighbor networks with significantly lower NODF values to be accepted. As the feasible set of networks grows exponentially with the number of pollinators, plants and interactions, choosing a cooling rate, α, and minimum temperature, T_{min}, that is sufficiently small becomes computationally impractical. We were able to achieve better results by reverting to the optimal solution we found so far during the algorithm in cases where the current solution had significantly lower nestedness values. To achieve this we compared the cost of the current network cost = 1−nodf(web) to the cost of the best network we observed so far $\text{cost}_{opt} = 1 - \text{nodf}(web_{opt})$ and reverted back to the optimal network with a probability given by $1 - e^{\frac{cost - cost_{opt}}{T}}$.

As a starting position we chose to proceed with the one produced by a modified version of the greedy algorithm proposed by Song *et al.* (2017). Our modification consists of adding a preference for links in the upper left positions of the biadjacency matrix, as these are more likely to enable further paired overlaps and decreasing marginal totals later in the simulation.

Further improvements were made to the algorithm by applying hill climbing each time a new optimal network was found by successively testing if a neighbor network is able to achieve a higher NODF value. If so, we proceed by choosing the neighbor that maximises the NODF score until no further neighbor network achieves a higher nestedness score than the current.

A fact that contributes to the potential of simulated-annealing algorithms is that the evaluating the NODF metric for neighbor networks can be implemented in a highly efficient way. As otherwise most of the computation time is spend evaluating the nestedness of neighbor networks we are able propose an algorithm that improves both the computational and the optimisation performance of the algorithm presented by Song *et al.*

Supplementary Figures

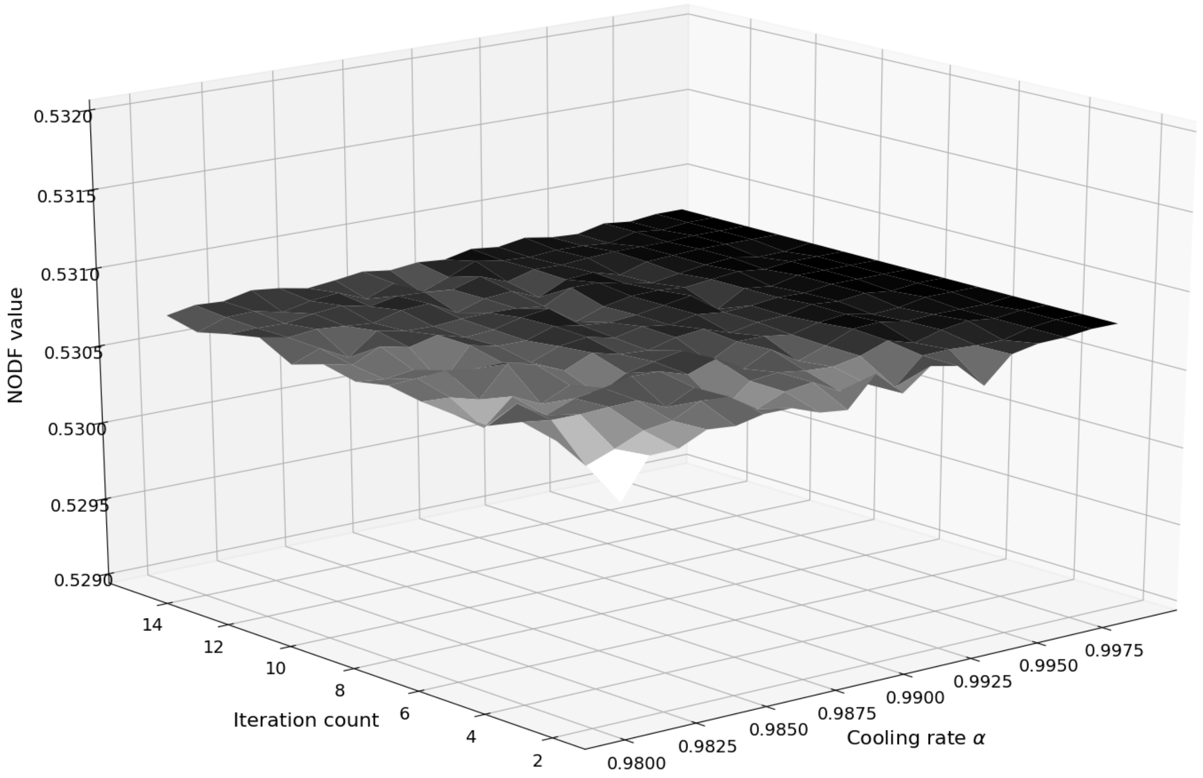

Supplementary Figure 1: The sensitivity of the simulated annealing algorithm to changes in parameter values for the network which achieved the largest increase in nestedness (17%) relative to the greedy algorithm. The y-axis gives the maximum NODF found by the algorithm, the x-axis gives the number of iterations run and the z-axis specifies the cooling rate. Changes in parameters have little effect on the maximum NODF value.

References

Song, C., Rohr, R. P., & Saavedra, S. (2017). Why are some plant–pollinator networks more nested than others? *Journal of Animal Ecology*, *86*(6), 1417–1424. doi:10.1111/1365-2656.12749
